# Proceeding abstracts: Erratum

**DOI:** 10.1097/MD.0000000000027190

**Published:** 2021-09-10

In “Proceeding Abstracts”^[1]^ which appeared in Volume 100, Issue 33 of *Medicine*, the title appeared incorrectly as “Proceeding Abstracts” and has been corrected to “4th Annual Mediclinic Middle East Research Conference.”
